# Diagnosis and management of acute infections during telehealth consultations in Australian general practice: a qualitative study

**DOI:** 10.3399/BJGPO.2023.0142

**Published:** 2024-03-20

**Authors:** Emma J Baillie, Gregory Merlo, Ruby Biezen, Kwame Peprah Boaitey, Parker J Magin, Mieke L van Driel, Lisa Hall

**Affiliations:** 1 General Practice Clinical Unit, Faculty of Medicine, The University of Queensland, Herston, QL, Australia; 2 Healthcare Improvement Unit, Queensland Health, Bowen Hills, Brisbane, QL, Australia; 3 Department of General Practice, The University of Melbourne, Melbourne, VI, Australia; 4 Institute for Evidence-Based Healthcare, Bond University, Gold Coast, QL, Australia; 5 School of Medicine and Public Health, University of Newcastle, Callaghan, NSW, Australia; 6 GP Training Research Department, Royal Australian College of General Practitioners, Callaghan, NSW, Australia; 7 School of Public Health, The University of Queensland, Brisbane, QL, Australia

**Keywords:** anti-bacterial agents, antimicrobial stewardship, general practitioners, qualitative research, referral and consultation, self-assessment, telemedicine

## Abstract

**Background:**

The use of telehealth has increased dramatically since the beginning of the COVID-19 pandemic. Little is known about how GPs manage acute infections during telehealth, and the potential impact on antimicrobial stewardship.

**Aim:**

To explore the experiences and perceptions of GP trainees’ and supervisors’ use of telehealth, and how it influences their management of acute infections.

**Design & setting:**

Australian GP registrars (trainees) and supervisors were recruited via email through their training organisations. Semi-structured interviews with 18 participants were conducted between July and August 2022.

**Method:**

Interviews were transcribed verbatim and analysed using a reflexive thematic approach.

**Results:**

We identified six overall themes. 1. Participants experienced impaired diagnostic capacity during telehealth consultations. 2. Attempts to improve diagnostic acuity included various methods, such as having patients self-examine. 3. The management of clinical uncertainty frequently entailed referring patients for in-person assessment, overinvestigating, or overtreating. 4. Antibiotic prescribing decisions during telehealth were informed by less information than were in-person consults, with varying impact. 5. Participants believed that other GPs improperly prescribed antibiotics during telehealth. 6. Supervisors believed that registrars hadn’t developed the knowledge or skills to determine when conditions could be managed appropriately via telehealth.

**Conclusion:**

Telehealth has potential for reducing transmission of acute infections and increasing access to healthcare. However, the implications of GPs, especially less experienced GPs, making diagnoses with less certainty, and consequently compromising antimicrobial stewardship, are a concern. Patient self-assessment tools may improve outcomes of telehealth consultations for acute infections.

## How this fits in

Telehealth consultations are now commonplace in general practice, and previous literature has investigated GPs’ attitudes towards their use. However, no literature has explored the influence that telehealth may have on the diagnosis and management of acute infections, nor its effect on antimicrobial stewardship. This study reports that acute infection diagnoses are made with less certainty during telehealth, and the various strategies that GPs use to manage this uncertainty. Furthermore, we found that telehealth has the potential to both protect and compromise antimicrobial stewardship.

## Introduction

Telehealth consultations have dramatically changed general practice consultations since their widespread uptake in 2020 due to the COVID-19 pandemic.^1^ Numerous countries have made permanent funding and regulatory changes to telehealth in general practice, which did not exist before the pandemic.^1^ Australia is one of those countries, retaining the inclusion of telehealth items on the Medical Benefits Scheme (MBS) post-pandemic.^2,3^ Telehealth is defined as a consultation via phone calls or video conferencing,^4^ not including asynchronous or text-based consultations. By May 2022, 95% of the Australian population had been vaccinated against COVID-19,^5^ but in what has been dubbed the ‘post-pandemic era’, consultations for acute infections are still being managed via telehealth, especially in practices where GPs avoid seeing patients with respiratory illnesses face-to-face. Since the beginning of 2021, the proportion of telehealth consultations has remained stable at approximately 20% of Australian GP consultations.^3^ The Royal Australian College of General Practitioners (RACGP) guidelines on managing acute respiratory illness recommend a telehealth-first approach to reduce disease transmission.^6^ The RACGP general guidelines for conducting telehealth consultations state that GPs should determine if physical examination is necessary when deciding to use telehealth services.^7^

Meanwhile, antibiotic resistance continues to be a global threat to health, exacerbated by unnecessary antibiotic prescribing.^8,9^ Prescribing antibiotics for acute respiratory infections is often considered unnecessary or inappropriate due to substantial evidence that they provide little-to-no benefit .^10^

Two systematic reviews have concluded that there is a lack of evidence for a relationship between antibiotic prescribing and any form of remote consultation.^11,12^ However, one systematic review found higher prescribing for otitis media and pharygnitis during telemedicine consultations.^13^ A recent Australian study reported an increase in antibiotic prescribing for respiratory infections during telehealth consultations, from 40.8% (95% confidence interval [CI] = 37.2 to 44.4) in 2020 to 61.0% (95% CI 59.1 to 63.0) in 2021, while face-to-face prescriptions remained stable.^14^

Qualitative evidence for the reasons why GPs prescribe antibiotics inappropriately during in-person consultations is well established. Rose *et al* outlined these factors, including GPs’ knowledge and attitudes towards antibiotic resistance, and non-clinical reasons, including patient pressure, time pressure, and financial incentives.^15^ Previous qualitative research examining GP registrars (trainees), found that influences to prescribe antibiotics inappropriately included diagnostic uncertainty, transitioning from hospital practice (where they see more severe infections), and their supervisors’ habits.^16^ Researchers have adapted the Centre for Disease Control antimicrobial stewardship (AMS) guidelines^17^ to create an AMS framework for telehealth, identifying barriers that physicians face during telehealth.^18^ However, this framework relies upon barriers which apply primarily to e-visits (virtual, text-based, and asynchronous consultations).^18^ No qualitative research on telehealth has specifically addressed GPs’ experiences with managing acute infections, or its perceived impact on antibiotic prescribing.

A GP demographic of particular interest in regard to AMS is GP trainees (called 'registrars' in Australia). These practitioners are establishing what will likely be persisting practice patterns.^19,20^ In addition, much of the current cohort of GP registrars in Australia (medical practitioners undergoing two years of specific postgraduate training to become specialist GPs) have undertaken their general practice based training wholly after the start of the pandemic. It is unknown how telehealth affects this group, and, given the pre-existing effect of diagnostic uncertainty on registrars’ antibiotic decision-making^16^ prior to the uptake of telehealth, understanding the impact of telehealth may be important for future antimicrobial stewardship.

The aim of this study is to explore the attitudes and perceptions of Australian GP registrars and their supervisors regarding the impact of telehealth consultations on their diagnostic processes and management of acute infections (especially their antibiotic prescribing).

## Method

### Setting and recruitment

Participants were Australian GP registrars or supervisors. Postgraduate GP vocational training in Australia consisted of a programme coordinated by geographically defined, not-for profit Regional Training Organisations (RTOs). Registrars complete three 6-month placements (training terms) in community general practice.^21^ During these training terms, registrars practice with considerable autonomy (but with access to clinical supervisors for guidance) in an apprenticeship-like model.^21^ They have prescribing rights identical to established GPs.

Participants were recruited via email, sent from a single RTO, encompassing New South Wales and the Australian Capital Territory. Emails were sent in May 2022 inviting study participation. Purposive sampling sought a maximum variation sample on the basis of gender, training term, supervisor experience, and rural or urban practices. GPs were provided with participant information sheets and consent forms prior to participation.

### Data collection process

Semi-structured interviews were conducted by video conferencing (Zoom) or telephone. A pilot interview with a GP registrar was performed to validate the interview schedule, and pilot data were not included in the analysis. A demographic questionnaire was administered at the start of each interview (Supplementary File S1). Interviews were participant-led as far as practicable, with participant responses informing further discussion.^22^

The interview schedule followed a theme list reflecting the study aims (Supplementary File 1). Questions were open-ended and designed to elicit participants’ experiences of using telehealth for acute infection consultations, diagnostic processes, and their perception of its impact on their prescribing. Participants were asked how lack of physical examination was compensated for in telehealth consultations. Interviews were audio-recorded and transcribed verbatim, both manually and using transcription software. Interviews were conducted until thematic saturation was achieved; that is, when no new meaningful themes are identified by the researchers.^23^ Data collection and analysis were iterative and cumulative.

### Analysis

Transcribed data were coded and analysed using the reflexive thematic approach.^24^ Coding was performed independently by EB, GM, and KPB, using NVivo (version 12) software. GM and EB coded transcripts and compared initial codes, then EB and KPB coded additional transcripts and checked for missing codes. Coding was consolidated at a third iteration, and recurring and/or related codes were mapped into themes. Functional and personal reflexivity was discussed at this stage of the analysis.^22^

Themes were analysed and codes were checked to ensure they appropriately reflected the data. Additionally, two experienced GP investigators, MvD and PM, provided a clinical review of transcripts and themes to determine if contextual GP nuances were accurately represented.

## Results

Seventy-two responses were received to the study invitation, and of these, nine supervisors and nine registrars were interviewed. Their demographic information is presented in Table 1. Supervisors’ experience ranged from 4 to 24 years; mean post-fellowship experience was 10 years. Registrars were mostly in Term 1 or Term 3. Interviews were conducted between June and July 2022, 2 years after MBS funding of telehealth commenced. At this time, a majority of consultations had returned to face-to-face with telehealth available as a permanent feature in Australian general practice,^3^ although continuing pressures caused by GP shortages should be noted.^25^ Most participants reported using telehealth as the main consultation mode intra-pandemic, but much less since. Telephone was unanimously the main mode of telehealth consultations.

**Table 1.** Participant demographics

Code^a^	Gender	Age, years	Years of experience ortraining term (T)	SEIFA^b^	Sessions per week
**S1**	F	30–34	<5 years	5	6
**S2**	M	40–44	5–10 years	2	9
**S3**	F	50–54	>20 years	4	4–5
**S4**	F	35–39	5–10 years	5	7–9
**S5**	F	35–39	5–10 years	4	6–7
**S6**	F	40–44	11–20 years	3	9
**S7**	F	35–39	5–10 years	2	8
**S8**	M	30–34	<5 years	4	8
**S9**	F	30–34	5–10 years	1	11
**R1**	F	25–29	T3	1	8
**R2**	M	45–49	T4	5	8
**R3**	F	25–29	T3	2	9
**R4**	M	25–29	T1	2	9
**R5**	F	25–29	T1	4	6
**R6**	F	30–34	T1	5	10
**R7**	F	25–29	T3	3	4
**R8**	M	25–29	T1	1	8
**R9**	F	25–29	T3	5	4

SEIFA = Socio-Economic Indexes for Areas.

^a^S = Supervisor. R = Registrar. ^b^Uses the Index of Relative Socio-economic Disadvantage (IRSD), where 1 indicates high socioeconomic disadvantage.

### Pre-consultation processes

Practices commonly used receptionists or booking platforms to screen patients for respiratory symptoms or triage them to determine if telehealth was appropriate. This was to ensure either that patients with respiratory illnesses had a telehealth consultation first (to avoid infection transmission risk) or, in contrast, to ensure patients with acute infections were seen face-to-face so they could be examined appropriately:

*‘Our reception would ask questions, “Do you have any symptoms of running nose or COVID symptoms?” They answer yes. They will often ask them not to come into the practice, to either take a phone call or telehealth consult first. Normally that’s pre-identified and a part of our booking system.’* (Supervisor [S]2, male)

*‘I told the receptionist if they have this issue* [an acute infection]*, just make sure they have a face-to-face appointment instead of telehealth.’* (Registrar [R]3, female)

Several participants felt that patients themselves tended to self-triage their consultation modality: the mildly unwell elected for telehealth consultations — usually for medical certificates — whereas more worried patients tended to choose face-to-face.

### Overall themes

We identified six key themes, presented in Table 2.

**Table 2.** Overall themes and subthemes

Theme	Subthemes
Impaired diagnostic capacity	1.1 Influence on diagnostic certainty 1.2 Patient familiarity impacted diagnostic confidence
Strategies to improve diagnostic acuity	2.1 Patient self-examination 2.2 Visual cues 2.3 Practitioner skills
Management of clinical uncertainty	3.1 Refer for in-person assessment 3.2 Overinvestigate 3.3 Overtreat
Changes to antibiotic prescription decision-making	4.1 Mixed effect on decision-making 4.2 Delayed prescribing 4.3 Patient demand for antibiotics
Perceptions about other GPs’ antibiotic prescribing	
Supervisor beliefs about their registrars	

### Theme 1 impaired diagnostic capacity

#### Subtheme 1.1. Influence on diagnostic certainty

All participants commented that the lack of physical examination during telehealth consultations impaired diagnostic capacity. Missing diagnostic information entailed missing visual cues, or missing the physical examination, leading to diagnoses or decisions made without the full clinical picture:

*‘I don’t doubt you’re certainly missing that eyeballing of a person, as to whether they look well or unwell … definitely there has to be an element that you’ve got a reduced diagnostic certainty over the phone, when you can’t see the patients. You can’t eliminate that factor.’* (S7, female)

However, this lack of physical examination affected participants differently. For some, among both registrars and supervisors alike, it created uncertainty, leading to *‘making decisions with half the information’* (R9, female):

*‘You can’t examine them and that’s a big part of our diagnostic process. So without that, it sometimes can be quite challenging.’* (R9, female)

For other participants, this lack of information didn’t greatly affect confidence in decision-making:

*‘It hasn’t been a barrier really, maybe because I’ve been lucky, again, whatever 80% of the time 90% of the time it’s a viral infection anyway, so I wouldn’t say it’s hindered my diagnostic abilities that much.*’ (R5, female)

#### Subtheme 1.2. Patient familiarity impacted diagnostic confidence

Telehealth MBS rebates are restricted to patients who have had previous in-person consultations. However, participants described situations where they still had encounters with new patients through telehealth, such as when the patient is familiar to the practice but not the practitioner, or patients who had been vaccinated at the practice and therefore qualified for a telehealth consult. For most of the participants, knowing the patient was integral to how confident they were with their diagnosis and management of acute infections. Conversely, new patients were considered much more challenging for diagnosing infections:

*‘I can’t even picture who am I dealing with. I find that that’s really hard.’* (R3, female)

*‘Because I know them quite well and because they’re my regular patients, I can usually pick up how they’re feeling.’* (S1, female)

### Theme 2: Strategies to improve diagnostic acuity

#### Subtheme 2.1: Patient self-examination

Without visual information or physical examination, almost all participants reported asking patients to perform aspects of a clinical assessment. This included a range of activities, broadly categorised into gathering clinical observation data (such as pulse rate or temperature) and carrying out their own physical examination (such as feeling lymph nodes):

*‘I think there’s other ways around it. If they have a UTI* [urinary tract infection]*, I can be like, “Look can you press on your tummy like over where your bladder is, can you examine yourself?”.’* (R7, female)

*‘So, I just try and be creative and I can also say like, “Google this particular condition and see what that looks like. Does that look like what you have?”’* (S9, female)

One participant, a supervisor, did not use any of the compensatory strategies used by other participants to address lack of physical examination:

*‘Uh, what do you mean? I mean, the same way as they tell me the case face to face. They tell me the story and I ask questions ... Look, maybe I potentially ask a few more questions, perhaps, a few more, things that I might not ask face to face. Um, yeah, I’m not sure else, how else otherwise.’* (S7, female)

#### Subtheme 2.2: Visual cues

Another method used to increase diagnostic information were requests for visual information: either for images to be sent or switching to video consultations, usually for conditions such as skin and soft tissue infections, or sore throat. This mitigated some of the information deficit, but had drawbacks such as patients not knowing how to take good images of skin conditions, older patients not being able to use technology:

*‘It’s a lot easier than not having the photo, because if you’ve got the photo there, you can ask the person, “Is it blanching, is it not? Does it feel hot?”. You have that visual cue to help guide you.’* (R6, female)

*‘Trying to get an 80-year-old to turn their video on, half your consult times gone, and the video is not even on.’* (S6, female)

#### Subtheme 2.3: Practitioner skills

Participants also adapted their own clinical skills to the consultation, such as taking a more thorough history and, mentioned with varying levels of certainty, was that they could determine severity of illness just by listening to a patient’s voice. Some felt this was intuitively gauged, confident with their impression of the patient, yet others thought this was a poor substitute:

*‘You can pick up on how someone’s feeling just by listening to their voice.’* (S1, female)

*‘In some ways you can get a nature of their voice and whether or not they’re struggling to breathe, that sort of stuff over the phone. But beyond that, I think it is difficult.’* (R9, female)

### Theme 3: Management of clinical uncertainty

If participants felt uncertain or that further information was needed before diagnosing or treating, they had three management pathways, as below.

#### Subtheme 3.1: Refer for in-person assessment

Participants could ask the patient to present in-person (either to the practitioner themselves, a colleague, an emergency department, or respiratory clinic):

*‘I feel like if I’m having a phone consult and I’m thinking, “Oh, I’m not quite sure, I’d really like to eyeball them” then I say, “Look, this probably isn’t suitable to continue as a phone consult. I’d really like to have a look, I need to have a listen to your chest or look in your ear, or whatever it might be.’* (S6, female)

#### Subtheme 3.2: Over-investigate

Participants could also order further investigation (chest x-rays, polymerase chain reaction tests, etc.):

*‘ … With my limitations over telehealth, I’m still trying to cover it up with these investigations.’* (S4, female)

#### Subtheme 3.3: Over-treat

Participants also reported overprescribing antibiotics:

*‘I do find myself reaching out for more antibiotics over the phone, than if I were to see the patient face-to-face. I think the fear is that I have missed something because I don’t have that physical examination with me.’* (R1, female)

### Theme 4: Changes to antibiotic prescription decision-making

#### Subtheme 4.1: Mixed effect on decision-making

Participants differed in their beliefs about the impact of telehealth on their antibiotic prescribing. Some felt more inclined to prescribe during telehealth consultations, while others refused to prescribe antibiotics at all (except for UTIs), and still others felt their management was unaffected. These differences were explained by several factors.

The higher inclination to prescribe was due to diagnostic uncertainty from the lack of clinical information. This was exacerbated if the participant believed that the patient would not be seen in-person, either because the patient was reluctant (due to vulnerability, difficulties leaving the house, or inconvenience, for example), or because the availability of in-person consultations was an issue:

*‘ … the less clear indications, the chesty cough, the earache, the sinus pain and whatnot, those things are really difficult. And I think probably myself and a lot of people would err on the side of caution and prescribe the antibiotics, whereas in person, we may have given a more watch-and-wait, sort of conservative approach to that.’* (S5, female)

*‘My feelings of uncertainty do play a part because I would rather cover my bases, if I haven't been able to see someone.’* (R4, male)

Participants who refuse to prescribe antibiotics via telehealth explained that, without a GP-performed physical examination, they couldn’t prescribe. These participants relied on patients presenting in-person, which was considered a viable management pathway. This was in direct contrast to those who felt more inclined to prescribe for whom not being able to see the patient often justified their antibiotic prescribing decisions:

*‘I can't complete my assessment unless you come in.’* (R8, male)

However, UTIs seemed to be the exception to this rule. Participants felt confident in prescribing empirically for UTIs, with the expectation that the patient would drop off a sample to pathology:

*‘Literally, other than UTI, I don't think I have done a single antibiotic prescription other than UTI without physically laying eyes on someone.’* (S6, female)

For those who believed that antibiotic prescribing was unaffected by telehealth, they either felt confident with their diagnosis and antibiotic prescribing decisions, or thought that the patient needed to be seen face-to-face to make a diagnosis:

*‘Most of the time, it’s they either need them or they don’t…Sometimes you just follow your gut, I don't think my gut would do different things over the phone if I’m not sure. I won't give them antibiotics just because I’m not sure, I’ll find another way to assess them a bit more.’* (R2, male)

#### Subtheme 4.2: Managing patient demand for antibiotics

Some participants mentioned that during telehealth it was easier to say ‘no’ to patient demands for antibiotics, justified by the lack of physical exam:

*‘I think telehealth may be easier to make excuse not to prescribe. You can always explain to the patient, I haven't seen it, I don't feel comfortable to prescribe you.’* (R3, female)

Conversely, others felt that lack of physical examination meant that explaining to the patient was more challenging:

*‘The patient has a more thorough experience of you laying on hands and examining them* [in-person]*, and they feel more thoroughly assessed, which increases their trust in you. Whereas over the phone, I think it’s harder to justify to the patient why you are or are not prescribing … I would be much more likely to give the antibiotics with a direct request over the phone, more so than I would be in person.’* (S5, female)

#### Subtheme 4.3: Delayed prescribing

Participant’s use of delayed prescribing during telehealth was considered to be similar to in-person consultations; it was used for upcoming weekends or holidays, if the patient was particularly anxious, or if there was established trust in the patient to use it wisely. Telehealth-related circumstances that prompted delayed prescribing included the patient being unable to come to the practice, and the increased desire for ‘safety netting’:

*‘I'm just more uncertain about what it could be. I don't have that full holistic assessment of them, like the vital signs or the physical examinations. So I think it’s* [delayed prescribing] *just a way of safety netting myself really.’* (R1, female)

### Theme 5: Perceptions about other GPs’ antibiotic prescribing

Participants who felt unaffected by telehealth voiced concerns about inappropriate practices by other GPs during telehealth. These concerns included assumptions that other GPs likely prescribe more antibiotics, prescribe without an indicated in-person examination, or prescribe antibiotics for confirmed COVID-19 infections:

*‘I'll see kids with respiratory symptoms and they'll say, “Oh, I've already done a telehealth with my GP and they've sent me a script for amoxicillin”, which I find really poor medicine, ’cause you know, they haven't even examined the child.’* (S9, female)

*‘I try to do the same antimicrobial stewardship that I would do in person. But for other colleagues, I think they're probably slightly more likely to prescribe.’* (S7, female)

### Theme 6: Supervisor beliefs about their registrars

Supervisors believed their registrars lacked the experience to judge appropriateness of over-the-phone infection management. Some mentioned their own confidence when judging if telehealth is an appropriate management pathway, but had to give guidance or ‘rules’ to their registrar who hadn’t yet acquired this knowledge:

*‘I've been a doctor for a long time. I think it probably has not affected my diagnostic skills and that I've been able to triage “No, you need to go get an examination ’cause you’ve got a red flag”, as opposed to, say, a registrar for example, who might try and do the whole thing on the phone and not actually know where you've gotta draw the line.’* (S3, female)

*‘My biggest challenge with telehealth wasn’t about me…it was about supporting my registrar who had never done telehealth before … he was already a fairly early term registrar anyway, and then on top of that, we’re chucking in a completely new system. So we had some fairly strict rules to begin with.’* (S6, female)

In their minds, this also impacted the GP registrars’ ability to hone their diagnostic skills and their antibiotic prescribing decision-making:

*‘I think they need to be seeing them more often than I do, ’cause they're inexperienced ... I think definitely, registrars, they shouldn't be giving antibiotics over the phone. That’s not a beneficial learning process for them if they are.*’ (S7, female)

## Discussion

### Summary

This study explored the attitudes and perceptions of GP registrars and supervisors of the impact of telehealth consultations on their diagnostic processes and management of acute infections. We found that both inexperienced GP registrars and experienced supervisors felt they were missing important diagnostic cues during telehealth consultations, and used varying strategies to gather more information. If this caused uncertainty, they felt the need to compensate by overprescribing antibiotics, ordering more investigations than usual, or referring patients for in-person examination.

Antibiotic prescribing, and thus stewardship, was affected both positively and negatively by telehealth. Several participants felt a higher likelihood to prescribe, and for those who felt no impact on their own prescribing, discussed instances of GP peers doing ‘inappropriate’ things (that is, prescribing without adequate examination). Antibiotic prescribing decisions were based on less information, which may further impact antimicrobial stewardship. Conversely, telehealth also influenced antimicrobial stewardship positively in some circumstances, with some participants finding it easier to deny requests for antibiotics for acute self-limiting infections via telehealth.

### Strengths and limitations

Themes were consistent across both types of participant, with the exception of Theme 6 (supervisor beliefs about their registrars). Nevertheless, the findings of this study may not be generalisable to other GPs, as our population of interest was GP trainees and supervisors. These practitioners may be particularly mindful of antimicrobial stewardship; this is supported by evidence that GP trainees and training practices have lower antibiotic prescribing than other GPs.^26,27^

The memory of the recent COVID-19 lockdowns is also a point of consideration, as limited in-person contact with patients may have affected their attitudes towards telehealth management. However, at the time of this study, the population in Australia had been vaccinated, and everyday life had largely returned to normal: no COVID-19 safety measures remained, face-to-face GP consultations were available, and international travel restrictions had been removed in March 2022.^28^ The proportion of telehealth consultation has also remained steady for 2 years, and as such, our findings are more generalisable to usual practice than if the study had been conducted earlier in the pandemic.^5,29^

The researchers include GPs and pharmacists who are actively involved in research, education, and clinical practice related to antimicrobial stewardship. The researchers have considered this in their reflexive thematic approach. Our analysis was further strengthened by the clinical review of transcripts and themes by the experienced GP investigators.

### Comparisons with existing literature

Our findings are consistent with a UK qualitative study investigating COVID-19 and antibiotic prescribing, which found that GPs perceived respiratory infections challenging to manage via remote consultations, but felt that UTIs were easily managed.^30^ All participants in our study reported that the decision to prescribe antibiotics for UTIs was easier than other conditions. Other research has comparably reported that the ability to see patients using video conferencing mitigates reduced diagnostic capacity by providing visual cues;^31^ however, video conferencing is seldom used, comprising less than 5% of GP telehealth consultations in Australia.^32^ To our knowledge, no other studies have reported the other strategies GPs implement to overcome telehealth’s limitations, nor its effect on antibiotic prescribing.

Participants in our study reported using delayed prescribing during telehealth, particularly when there was an upcoming weekend or holiday, if there was clinical uncertainty to ‘safety net’ themselves, or if the patient was particularly anxious. These circumstances for initiating a delayed prescription are consistent with the literature on face-to-face consultations,^33,34^ with the added potential for reduced clinical certainty during telehealth from the lack of physical examination.

A rapid review found the loss of physical and visual assessments during telehealth to be an issue, consistent with our participant attitudes.^35^ These concerns are not unfounded; an Australian coroner’s inquest investigated a telehealth consultation involving patient with self-diagnosed gastroenteritis with GP-guided self-examination.^36^ The misdiagnosed bowel obstruction was fatal.^36,37^ A GP in our study also reported asking patients with UTIs to self-palpate their abdomen, and although the coronial case represents a worst case scenario, the need for physical examination is paramount for certain diagnoses. On the other hand, other research has shown that patients may be capable of doing their own assessment for sore throat, if given appropriate tools and guidance.^38^

### Implications for research and practice

Telehealth has undoubtedly increased access to healthcare for patients, enabled better continuity of care for the management of chronic disease, and reduced infection transmission.^39–41^ However, in the context of acute infections, telehealth may be considered a balancing act. The potential to reduce infection risk, and protect vulnerable patients must be weighed with impact on antimicrobial stewardship, reduced diagnostic certainty, possible delays in appropriate treatment and, conceivably, poorer patient outcomes. GPs need further training and condition-specific recommendations during telehealth, combined with self-assessment tools for patients.

The need for a physical examination, and lack of a GP–patient relationship, are consistently cited as situations where telehealth is considered inappropriate;^7^ however, in our study, GPs describe ongoing telehealth use in both of these circumstances. Policymakers should be made aware of these occurrences.

Furthermore, GP trainees lack experience judging when an in-person consultation is required, and are still developing their persisting prescribing habits under the guidance of their supervisors in this new context. GP registrars and supervisors require additional support navigating the appropriate use of telehealth for acute infections to preserve antimicrobials for the future.

We urge educators and researchers to develop and implement patient self-assessment tools, so that more reliable diagnostic information is available to GPs during telehealth consultations. Additionally, when considering the need for antibiotics, GPs should consider physical examination to complete their diagnosis for acute infections.

## Data

The dataset is not publically available under the HREC approval requirements.
